# Effects of combined aerobic-resistance training on health-related quality of life and stress in sedentary adults

**DOI:** 10.3389/fragi.2025.1603635

**Published:** 2025-08-18

**Authors:** Fernanda M. Silva, José P. Ferreira, Ana M. Teixeira, Alain Massart, Pedro Duarte-Mendes

**Affiliations:** ^1^ Faculty of Sport Sciences and Physical Education, University of Coimbra, CIPER, Coimbra, Portugal; ^2^ School of Education and Communication, University of Algarve, Faro, Portugal; ^3^ Department of Sports and Well-being, Polytechnic University of Castelo Branco, Castelo Branco, Portugal; ^4^ Sport Physical Activity and Health Research & Innovation Center (SPRINT), Castelo Branco, Portugal

**Keywords:** psychological stress, wellbeing, HRQOL, physical activity, sedentary behavior

## Abstract

**Introduction:**

Poor quality of life and psychological stress have been associated with worse clinical outcomes, including anxiety and depression disorders, cardiovascular diseases, metabolic syndrome, type 2 diabetes, and premature mortality. Evidence suggested that physically active adults were more likely to report better quality of life and increased capacity to deal with stress, compared to their sedentary peers. This study examines the effects of 16 weeks of combined aerobic and resistance exercise training on health-related quality of life (HRQoL) and stress levels in sedentary adults.

**Methods:**

This study involved previously sedentary middle-aged workers (n = 36), randomized, and allocated into control (n = 18) and exercise (n = 18) groups. The exercise group performed 16 weeks of combined exercise training for 75 min, three times/week. The HRQoL was assessed using the SF-36 survey and Satisfaction with Life Scale. Stress levels were assessed subjectively using the Perceived Stress Scale and objectively by the salivary cortisol and alpha-amylase concentrations. Intra- and inter-group analysis were performed using a mixed ANOVA or Friedman’s test.

**Results:**

In relation to SF-36 results, a significant improvement in the mental component summary (*p* = 0.047, Kendall’s W = 0.170 (small effect)) and social functioning subdomain (*p* = 0.040, Kendall’s W = 0.179 (small effect)) was found for the exercise group after the intervention. A significant difference between groups was found in mean differences (Δ) in the mental health subdomain, with superiority in the exercise group (exercise group, Δ = 7.50 vs. control group, Δ = −5.00; *p* = 0.006). After 16 weeks of follow-up, the exercise group perceived reduced stress levels compared to the control group (exercise group, Δ = −3.67 points vs. control group, Δ = 0.94 points; *p* > 0.05); although not significant, this result is clinically relevant. The exercise program had no significant effect on salivary stress biomarkers.

**Discussion:**

The present study’s findings may have important clinical implications because they show that middle-aged sedentary workers are likely to benefit from adopting a regular combined exercise training regimen to promote better HRQoL (i.e., mental health component) and wellbeing.

**Clinical Trial Registration:**

clinicaltrials.gov, identifier NCT04868240.

## 1 Introduction

Sedentary behavior–characterized by waking behaviors in a sitting, reclining, or lying posture and energy expenditure ≤1.5 metabolic equivalents (METs) (Tremblay et al., 2017) – has been associated with several adverse health outcomes including obesity, metabolic syndrome, type 2 diabetes (T2D), cardiovascular diseases, and mortality, independently of physical activity levels (Davi et al., 2018; Patterson et al., 2018; Ekelund et al., 2019). A growing body of evidence has also suggested that prolonged sedentary behavior may negatively impact health-related quality of life (HRQoL) (Kim et al., 2017; Kolt et al., 2017), capacity to cope with stress (Gilson et al., 2017; Gubelmann et al., 2018) and sleep quality (Koohsari et al., 2023; Liangruenrom et al., 2023). These domains have been increasingly acknowledged as important health outcome measures.

HRQoL is a multidimensional concept encompassing physical, emotional, mental, and social wellbeing (Collins et al., 2021). Reduced HRQoL has been associated with worse clinical outcomes, such as hospitalization and premature mortality (Phyo et al., 2020). Psychological stress is a negative emotional state that may cause a variety of negative conditions, including poor mental health (O’Connor et al., 2021), increased risk of anxiety and depression disorders (Wiegner et al., 2015), sleep problems (Chen et al., 2023), cardiovascular diseases (Sara et al., 2018), metabolic syndrome and type 2 diabetes (T2D) (Harris et al., 2017), and premature death (Prior et al., 2016). Due to their convenient collection and analysis, salivary biomarkers are easily included in neuroendocrine (stress) research (Strahler et al., 2016). Among these, salivary cortisol has been routinely used as a biomarker of psychological stress and related physical and mental conditions (Hellhammer et al., 2009). It is considered a reliable measure of the hypothalamic-pituitary adrenal axis (HPA) adaptation to stress (Hellhammer et al., 2009). In addition, salivary alpha-amylase activity has been recognized as a reliable and rapid biomarker for measuring response to stress across different clinical settings, including those in psychology and neuroscience (Aita et al., 2024). This enzyme is recognized as a valid and reliable marker of autonomic nervous system (ANS) (dys)function in stress-related research (Strahler et al., 2016; Ali and Nater, 2020). Importantly, the European Agency for Safety and Health at Work found that 59% of Portuguese workers indicate that stressful situations are common in their workplaces (European Agency for Health and Safety at Work, 2013).

Most working adults spend approximately 8 h a day at work, so occupational tasks and work settings may significantly impact their movement behaviors (Prince et al., 2019). A review found that working adults spent about 60% of their working and waking time sedentary, and only 4% of the day included physical activity of moderate-to-vigorous intensity (Prince et al., 2019). Furthermore, the desk-based workers revealed the greatest time in sedentary behaviors and the lowest time in physical activity (Prince et al., 2019). The authors suggested that effective interventions that promote health-enhancing physical activity are needed to mitigate the harmful impact of sedentary behavior among workers (Prince et al., 2019).

Regular exercise has been recognized as a promising non-pharmacological strategy for preventing and treating many health issues (Pedersen and Saltin, 2015). Studies showed that physically active adults were more likely to report better HRQoL (DiPietro et al., 2019), satisfaction with life (Zayed et al., 2018), and increased capacity to deal with stress (Gubelmann et al., 2018; Scully et al., 1998) compared to their sedentary peers. Current evidence from clinical trials indicates that participation in an exercise program may have a beneficial effect on HRQoL domains (Collins et al., 2021; Pietta-Dias et al., 2019). In turn, the evidence about the impact of an exercise program on perceived stress is poorly studied, evidencing either conflicting or null exercise effects (Carraça et al., 2021). Among the different exercise modes, the World Health Organization (WHO) recommends that adults accumulate an average weekly volume of 150–300 min of moderate-intensity or 75–150 min of vigorous-intensity and two or more days a week of muscle-strengthening activities at moderate or greater intensity for greatest health improvements (Bull et al., 2020). This is consistent with a previous study conducted on healthy older adults that found that combined aerobic and resistance training resulted in higher amelioration in quality of life compared to aerobic or resistance training alone (Pietta-Dias et al., 2019). Similarly, Collins et al. (2021) also found that 8 months of combined exercise training had the greatest improvement in physical component score, mental component score, and individual subdomain scores of HRQoL compared with aerobic or resistance training alone. However, conflicting results were found in other studies. Tozetto et al. (2022) found that a fixed intensity combined training was effective in improving the mental component score but not the physical component in adults with obesity. Similarly, Sillanpää et al. (2012) found an improvement in general health (subdomain of physical component) and a tendency to worsen the domains of role-physical and bodily pain in sedentary middle-aged and older adults. The experimental studies assessing salivary alpha-amylase and cortisol measurements in the context of exercise training are still scarce and present inconsistent results. A study (Klaperski et al., 2014) investigated the impact of a 12-week exercise training program on physiological stress response to a psychosocial stressor and found that exercise training significantly improved fitness and reduced stress reactivity, measured by cortisol, heart rate, and heart rate variability. In turn, a study in institutionalized older women (Rieping et al., 2019) found that 14 weeks of chair-based aerobic training and chair-based elastic-band strength training promoted a slight (but non-significant) increase in alpha-amylase and cortisol levels.

Evidence about the effects of combined aerobic and resistance training on HRQoL domains and stress is insufficient and controversial in literature. Therefore, this study aimed to evaluate the effects of 16 weeks of combined training on the HRQoL and stress levels (assessed by perceived stress and salivary biomarkers) in sedentary middle-aged workers. Considering that combined exercise training enhances parameters related to health in sedentary adults, it was hypothesized that this program would improve the HRQoL domains and reduce participant’s stress levels.

## 2 Methods

### 2.1 Study design

This randomized controlled study adheres to the Declaration of Helsinki, was approved by the Ethical Committee for Health (CE/FCDEF-UC/00512019) of the FCDEF, University of Coimbra, and is registered on Clinicaltrials.gov (NCT04868240). The assessments were conducted at baseline, as well as 8 and 16 weeks after the intervention. Methodological details of this trial can be found in the study protocol article (Ferreira et al., 2022).

### 2.2 Participants

Full-time working adults aged 40–64 years old were recruited from different companies. Eligibility criteria were adults working in sedentary jobs (i.e., report spending ≥65% of their working hours sedentary), not engaging in regular exercise in the past 6 months, having no physical disability that precluded participation in the intervention, no history of major chronic diseases (i.e., cancer, cardiovascular or metabolic disease), no uncontrolled blood pressure, no major depressive or cognitive disorder (Ferreira et al., 2022). The sample size was calculated *a priori* using the G*Power Software (version 3.1.9.2, University of Kiel, Germany) by setting the test family as “*F tests*” and statistical test “*ANOVA: Repeated Measures, within-between interaction*.” Adopting an interaction Cohen *d* = 0.25, calculated based on the primary outcome of this project (i.e., HOMA-IR), 36 participants were needed to achieve an 80% statistical power (α = 0.05). After signing the free and informed consent form, participants performed a set of assessments and were posteriorly randomized and allocated into two groups: the control group and the exercise group. The allocation of the participants was concealed from the assessment staff. The control group did not receive any intervention, whereas the exercise group participated in a supervised combined training program. Participants who decided to withdraw from the study or who changed their daily living activities and dietary habits were excluded from the analysis.

### 2.3 Intervention

Participants in the exercise group attended a 16-week combined training program, with a weekly frequency of three times a week, and a duration of 75 min per session (i.e., achieving the 225 min/week recommended by the current physical activity guidelines) (Ferreira et al., 2022). The first part of the session was reserved for resistance training followed by aerobic training. For resistance exercise training, participants performed exercises involving the use of body weight and free weights as described elsewhere (Ferreira et al., 2022), with a prescription progressing between light-to-moderate and moderate-to-vigorous intensities (45%–90% of predicted 1-repetition maximum) (Ferreira et al., 2022). For aerobic training, brisk walking and running, stepping or a circuit methodology were used, with the intensities prescribed based on maximum heart rate (HR_max_) (60%–95% HR_max_) (Ferreira et al., 2022). Both resistance and aerobic training were periodized, and the training loads and intensities increased consistently throughout the weeks (Ferreira et al., 2022). All exercise sessions started with 10 min of light-to-moderate intensity cardiorespiratory and muscular endurance warm-up and ended with 5 min of muscle relaxation (Ferreira et al., 2022). Training sessions were conducted in small groups and were delivered by two certified instructors with at least 3 years of experience. An adherence of at least 80% to the exercise program was required to a participant be included in the analysis.

### 2.4 Primary outcomes

#### 2.4.1 36-Item short form survey

The 36-Item Short Form Survey (SF-36) (Ware and Sherbourne, 1992), validated for the Portuguese adult population (Ferreira, 2000a; Ferreira, 2000b), was applied to evaluate HRQoL. This survey comprises 36 questions that cover eight subdomains of health, including (i) General health, (ii) Bodily Pain, (iii) Physical functioning, (iv) Role-physical, (v) Vitality, (vi) Role-emotional, (vii) Social functioning, and (viii) Mental health. These subdomains were grouped into two component scores: a physical component summary and a mental component summary. The scores for each subdomain and component range from 0 to 100, with higher values representing better HRQoL (Ware and Sherbourne, 1992).

#### 2.4.2 Satisfaction with life scale

The Satisfaction with Life Scale (SWLS) (Diener et al., 1985), validated for the Portuguese adult population (Neto et al., 1990), was applied to measure cognitive judgments of participant’s life satisfaction. This scale comprises five items formulated in the positive sense, with structured responses on a 5-point Likert scale (range from ‘strongly disagree’ to ‘strongly agree’). Higher scores represent higher life satisfaction.

#### 2.4.3 Perceived Stress Scale

The Perceived Stress Scale (Cohen et al., 1983), validated for the Portuguese adult population (Ribeiro and Marques, 2009), was also applied to assess the participant’s life situations considered as stressful during the previous month. This scale comprises 13 questions with structured responses on a 5-point Likert scale (ranging from never to very frequently). Items 4–7, 9, 10, and 13 were inverted to calculate the final score. Higher scores represent higher perceived stress levels (Cohen et al., 1983).

#### 2.4.4 Saliva samples

Saliva samples were collected to determine the salivary concentrations of cortisol and alpha-amylase. Participants sat quietly with their heads tilted slightly forward and eyes open and passively dribbled unstimulated saliva into a pre-weighted polypropylene tube for 4 min (Ferreira et al., 2022; Silva et al., 2022; Li and Gleeson, 2004). Participants were instructed to maintain a hydrated state and to avoid brushing their teeth for 1 hour, drink alcoholic drinks for 12 h, and consume high-acid or high-sugar food immediately before saliva collection (Ferreira et al., 2022; Silva et al., 2022). The saliva samples were subject to a single freeze-thaw cycle. On the day of the assay, saliva samples were placed into Eppendorf microcentrifuge tubes after thawing at room temperature and centrifuged at 13,000 rpm for 4 min to sediment solid particles and insoluble protein (Li and Gleeson, 2004). Salivary cortisol was determined using a commercially available high-sensitivity enzyme immunoassay kit (Salimetrics, State College, PA, United States). The lower sensitivity limit and range of detection limits for cortisol were <0.007 and 0.012–3.000 μg/dL. Salivary alpha-amylase was determined using a kinetic reaction assay (Salimetrics, State College, PA, United States) with a lower limit of sensitivity of 2.0 U/mL and an estimated absolute range of 3.1–423.1 U/mL. All saliva collections occurred at the same time in the morning and at ambient room temperature (24 °C–26 °C).

### 2.5 Secondary outcomes

Participants completed a questionnaire before the intervention, where they provided sociodemographic information, including age (complete years), sex (female or male), marital status (with or without partner), and education level. Body weight (kg) and height (cm) were determined through a calibrated digital scale (SECA 761, Germany) and a portable stadiometer (Seca Bodymeter 208, Germany), respectively. Waist and hip circumferences were measured using a flexible tape measure (Hoechstmass-Rollfix, Germany) according to the standardized procedures (National Center for Health Statistics U.S., 2007). Fasting blood samples were collected to determine glucose, total cholesterol, high-density lipoprotein (HDL-C), low-density lipoprotein (LDL-C), and triglycerides at baseline. Blood pressure was also assessed at baseline using an automated oscillometer cuff (Norav NBP-24 NG, Wiesbaden, Germany). These data were used for sample characterization.

### 2.6 Statistical analysis

Data are presented as mean ± standard deviation for continuous variables and as frequency and percentage for categorical variables. The Shapiro-Wilk and Levene tests were used to verify data normality and homogeneity, respectively. A Z-score dividing the skewness values or excess kurtosis value by their standard errors was also used to determine the statistical significance of any deviation from the normal distribution, with a z-score of ≤1.96 suggesting a normal distribution of the data (Field, 2018). Non-normal data were log-transformed. The absence of extreme outliers was guaranteed through standard visual inspection. Baseline differences between groups were determined by independent T-test for continuous variables and chi-squared or Fisher’s exact test for categorical variables. Intra- and inter-group analysis were performed using a mixed ANOVA for repeated measures for normally distributed data (i.e., the Satisfaction with Life Scale, Perceived Stress Scale, and the SF-36 subdomains: Physical Component Summary, General Health, Bodily Pain, and Vitality), with Bonferroni-adjusted post hoc tests. Friedman’s test was also used for data that were not normally distributed (i.e., SF-36 subdomains: Role Physical, Physical Functioning, Mental Component Summary, Role Emotional, Social Functioning, and Mental Health). When a significant overall effect was detected (*p* < 0.05), pairwise comparisons were conducted using the Wilcoxon signed-rank test. The multiple comparison test used the Bonferroni correction to avoid error Type I (Ho, 2014). The Mann-Whitney test was used to assess differences between groups in non-normally distributed data. The effect size for *F-statistics* was reported as partial eta-squared (ƞ^2^p), considering the interpretation as small (0.01–0.05), medium (0.06–0.13), and large (≥0.14) (Cohen, 1988). Therefore, Kendall’s W effect size (suitable for the Friedman test) was also calculated and interpreted as small (≥0.01), medium (≥0.3), and large (≥0.5) (Fritz et al., 2012). The magnitude of the post 8- and 16-week minus baseline difference was expressed as the mean difference (Δ). Statistical analysis was performed on SPSS Statistics version 27.0 (SPSS Inc., IBM Company, Chicago, Illinois, United States) and GraphPad Prism 9.0 software (GraphPad Software, San Diego, CA, United States). The analyses present were performed “per protocol”. Significance was accepted at *p* < 0.05.

## 3 Results

### 3.1 Descriptive statistics

**FIGURE 1:**
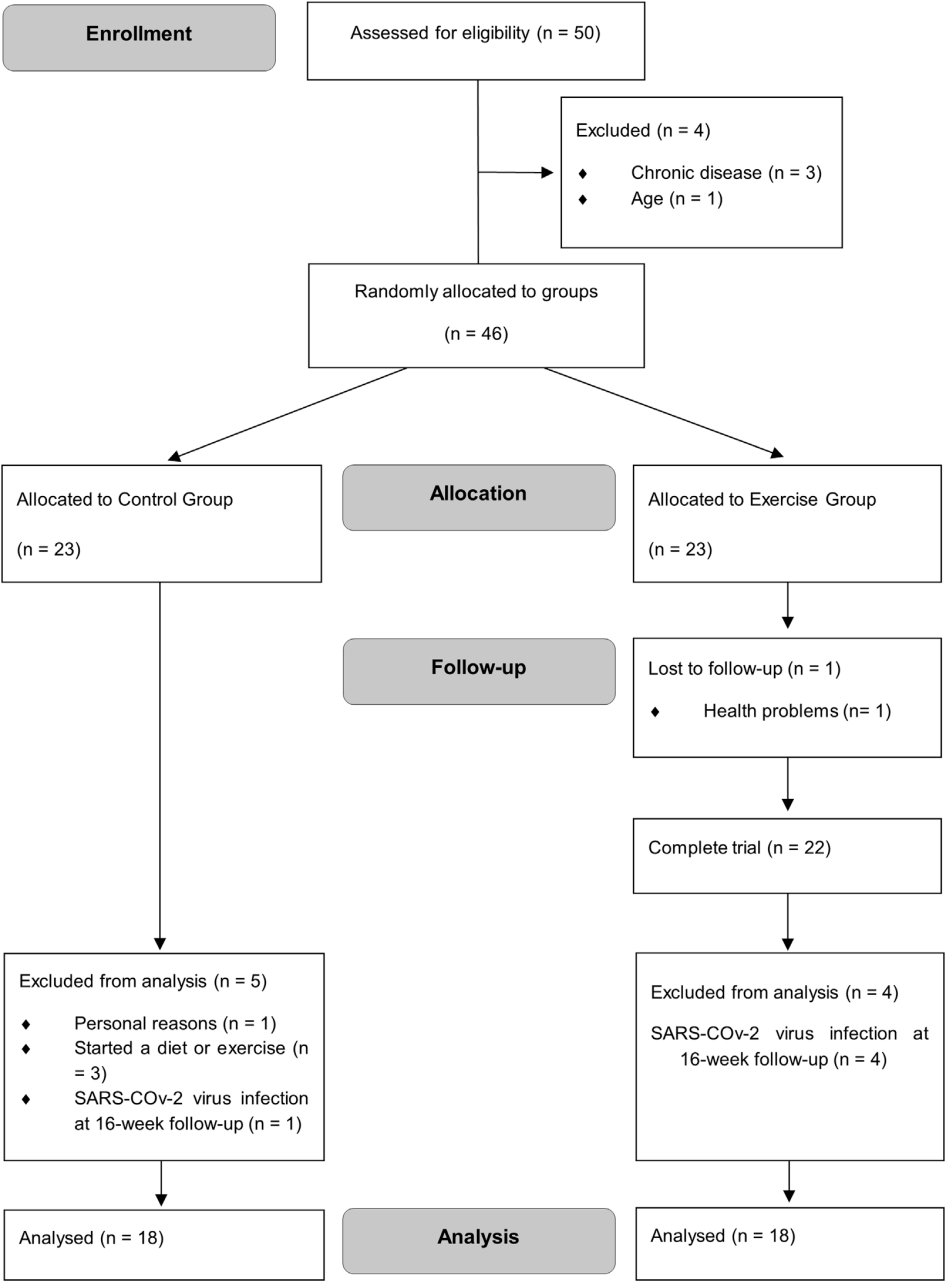
CONSORT trial flow diagram.

The study recruited 50 volunteers. After considering the eligibility criteria, 46 participants were randomized into two groups (control group, n = 23; exercise group, n = 23). A total of 36 participants completed the study and were included in the analysis (n = 18 in each group) (Figure 1).

Table 1 presents the baseline characteristics of the participants by group. No significant differences were found between the groups (*p* > 0.05) according to the analyzed variables. There were no major adverse events associated with study intervention and procedures. Participants of the exercise group reached 86.2% session frequency.

**TABLE 1:** Baseline characteristics of the participants who completed the trial.

Variables	Total sample (n = 36)	Control group (n = 18)	Exercise group (n = 18)	*p*
	Mean ± SD	Mean ± SD	Mean ± SD	
Age (years)*	53.70 ± 6.92	52.24 ± 7.84	55.15 ± 5.72	0.355
Height (cm)	161.01 ± 8.38	162.49 ± 7.84	159.54 ± 8.86	0.298
Body mass (kg)	70.98 ± 13.42	74.76 ± 14.05	67.20 ± 11.97	0.091
BMI (kg/m^2^)	27.24 ± 3.92	28.18 ± 4.17	26.31 ± 3.51	0.155
Waist circumference (cm)	96.62 ± 10.49	98.60 ± 10.97	94.64 ± 9.89	0.263
Hip circumference (cm)	104.39 ± 9.13	107.06 ± 10.62	101.73 ± 6.61	0.079
VO_2max_ (ml_o2_/kg/min)	33.13 ± 4.23	32.28 ± 4.44	33.97 ± 3.95	0.237
Systolic BP (mmHg)	119.33 ± 11.10	118.77 ± 12.21	119.90 ± 10.19	0.765
Diastolic BP (mmHg)	72.43 ± 8.68	72.13 ± 6.61	72.73 ± 10.55	0.839
Total cholesterol (mg/dL)^+^	201.06 ± 39.93	200.28 ± 41.13	201.83 ± 39.88	0.909
LDL-cholesterol (mg/dL)	119.53 ± 32.35	122.56 ± 30.21	116.50 ± 34.95	0.582
HDL-cholesterol (mg/dL)	62.46 ± 17.16	57.15 ± 13.00	67.78 ± 19.43	0.062
Triglycerides (mg/dL)^+^	92.86 ± 35.67	97.61 ± 36.13	88.11 ± 35.59	0.432
	*n* (%)	*n* (%)	*n* (%)	
Women	29 (80.6)	15 (83.3)	14 (77.8)	0.674
Marital status				
Single	2 (5.6)	2 (11.1)	0 (0)	0.506
Married/Living as a couple	22 (61.2)	11 (61.2)	11 (61.2)	
Divorced	12 (33.3)	5 (27.8)	7 (38.9)	
Education				
High school	13 (36.1)	6 (33.3)	7 (38.9)	0.529
Graduate degree	23 (63.9)	12 (66.7)	11 (61.1)	

Abbreviations: BMI, body mass index; VO_2max_, maximal oxygen uptake; BP, blood pressure; HDL, high-density lipoprotein; LDL, low-density lipoprotein; SD, standard deviation; n, absolute frequency; %, relative frequency; *Mann-Whitney test.

^+^ Indicate that statistical analysis was performed on transformed data due to non-normal distribution.

### 3.2 Exercise group significantly improved mental health component summary

**FIGURE 2:**
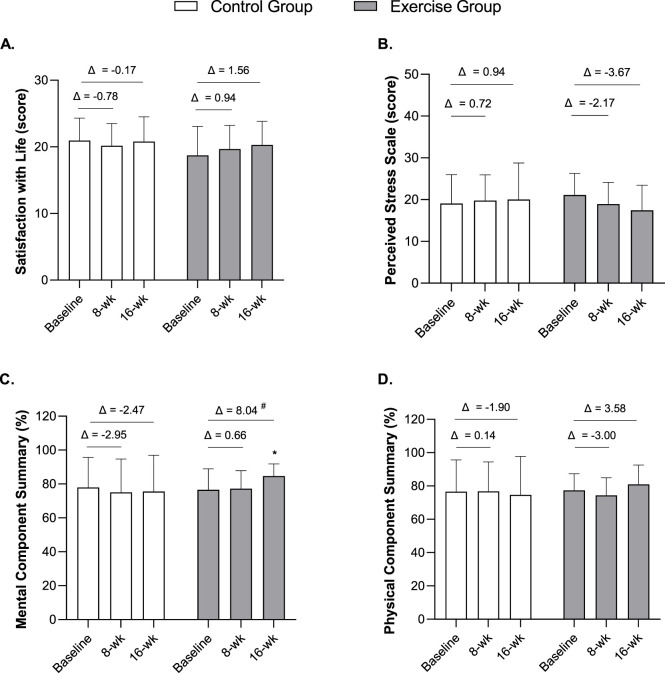
Mean scores in the Satisfaction with life scale **(A)**, Perceived stress levels **(B)**, SF-36 Mental Component Summary **(C)**, and the SF-36 Physical Component Summary **(D)** at baseline, 8 weeks, and 16 weeks in middle-aged adults. Notes: Data are expressed as mean ± standard deviation. Δ indicates the difference between post-8 or post-16 weeks and baseline. *A significant improvement was observed in the Mental Component Summary for the exercise group (*X* ^*2*^_*F*_ = 6.113, *df* = 2, *p* = 0.047, Kendall’s W = 0.170); Pairwise comparisons showed significant differences between baseline and 16 weeks (within-group) and between 8 and 16 weeks (within-group). ^#^ *p* < 0.05, significant difference in Δ (16 weeks–baseline) between groups by Mann Whitney test (*U* = 83.50, *z* = −2.484, *p* = 0.012). *n* = 36 (*n* = 18 in the control group, *n* = 18 in the exercise group).

The results of HRQoL’s main components, life satisfaction and perceived stress, are presented in Figure 2. Concerning satisfaction with life scale and perceived stress levels (Figures 2A,B, respectively), no significant intra- and inter-group differences were observed (*p* > 0.05); however, the exercise group showed a trend to increase their life satisfaction (exercise group, Δ = 1.56 points (95% CI -0.52, 3.63) vs. control group, Δ = −0.17 points (95% CI -1.76, 1.43)) and to decrease their perceived stress levels (exercise group, Δ = −3.67 points (95% CI -6.97, −0.364) vs. control group, Δ = 0.94 points (95% CI -2.57, 4.45)) after the 16-weeks of intervention.

In relation to the main components of the SF-36, a significant improvement was found in the Mental Component Summary for the exercise group after the exercise program (*X* ^*2*^_*F*_ = 6.113, df = 2, *p* = 0.047, Kendall’s W = 0.170 (small effect)) (Figure 2C). Pairwise comparisons revealed significant improvements between week 8 and week 16 (*p* = 0.006), as well as between baseline and week 16 (*p* = 0.012). Moreover, a significant difference between groups was found for mean changes (Δ 16-weeks - baseline) in the Mental Component (exercise group, Δ = 8.04 (95% CI 1.33, 14.75) vs. control group, Δ = −2.47 (95% CI -10.11, 5.18); *U* = 83.50, *z* = −2.484, *p* = 0.012).

The exercise group showed a trend to improve the Physical Component Summary (time*group interaction *F* = 2.932, *p* = 0.060, *ƞ* ^*2*^ *p* = 0.079 (medium)) after the 16-week follow-up; however, this effect was not significant (Figure 2D). Additional graphs illustrating individual-level trajectories and raw data for these outcomes are presented in the Supplementary Material (Supplementary Figure S1).

**FIGURE 3:**
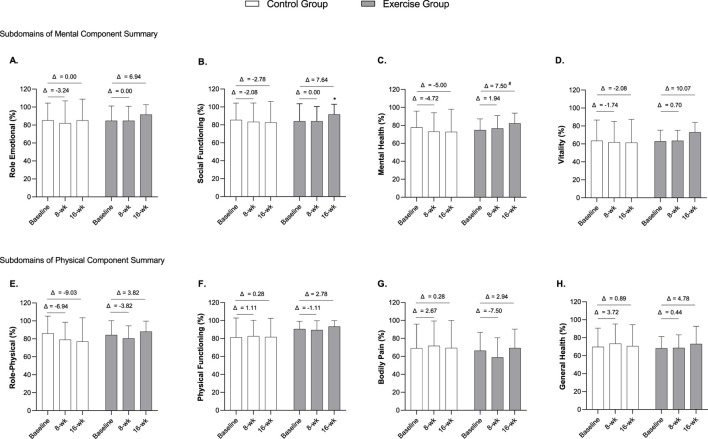
Differences between baseline and 8- and 16-week follow-up and between groups on SF-36 Mental Component subdomains (Role emotional **(A)**, Social functioning **(B)**, Mental health **(C)**, and Vitality **(D)**) and Physical Component subdomains (Role-physical **(E)**, Physical functioning **(F)**, Bodily Pain **(G)**, and General Health **(H)**. Notes: Data are expressed as mean ± standard deviation. Δ indicates the difference between post 8- or 16-week follow-up and baseline. *A significant improvement in Social Functioning subdomain was observed in the exercise group (*X* ^*2*^_*F*_ = 6.450, *df* = 2, *p* = 0.040, Kendall’s W = 0.179). ^#^ *p* < 0.05, significant difference in Δ (16-weeks–baseline) between groups by Mann Whitney test (*U* = 76.50, *z* = −2.764, *p* = 0.006). *n* = 36 (*n* = 18 in the control group, *n* = 18 in the exercise group).

In relation to the subdomains of Mental Component Summary (Figures 3A–D), a significant improvement in Social Functioning was observed for the exercise group throughout the intervention (*X* ^*2*^_*F*_ = 6.450, df = 2, *p* = 0.040, Kendall’s W = 0.179 (small effect); Figure 3B). However, pairwise comparisons revealed no significant differences between time points: baseline vs. week 8 (*p* = 0.904), baseline vs. week 16 (*p* = 0.094), and week 8 vs. week 16 (*p* = 0.053). Additionally, a significant difference between groups was found in change scores (Δ) in the mental health subdomain (exercise group, Δ = 7.50 (95%CI -0.33, 15.33) vs. control group, Δ = −5.00 (95%CI -13.49, 3.49); *U* = 76.50, *z* = −2.764, *p* = 0.006; Figure 3C). While no significant intra- and inter-group differences were observed for the remaining Mental Component subdomains (*p* > 0.05), the exercise group tended to improve their mean scores, while the control group worsened (Figures 3A,D). Additional graphs illustrating individual-level trajectories and raw data for these outcomes are presented in the Supplementary Material (Supplementary Figure S2). Furthermore, no significant changes or differences between groups were observed for the different Physical Component subdomains (*p* > 0.05; Figures 3E–H). Nevertheless, individual-level trajectories for these outcomes are also presented in the Supplementary Material (Supplementary Figure S3).

In relation to the salivary stress components (i.e., cortisol and alpha-amylase; Table 2), there were no significant effects of time, group, or interaction (*p* > 0.05).

**TABLE 2:** Differences between baseline and 16-week follow-up and between groups on salivary stress markers.

Variables	Control group (n = 18)			Exercise group (n = 18)			Time factor		Group factor		Interaction	
	Baseline	16 weeks	Δ (95%CI)	Baseline	16 weeks	Δ (95%CI)	*p*	*η* ^*2*^ *p*	*p*	*ƞ* ^*2*^ *p*	*p*	*η* ^*2*^ *p*
Salivary cortisol (µg/dL)	0.29 ± 0.14	0.29 ± 0.10	−0.005 (−0.10, 0.09)	0.34 ± 1.12	0.33 ± 0.12	−0.01 (−0.06, 0.04)	0.780	0.002	0.136	0.064^b^	0.852	0.001
Salivary α-amylase, (U/mL)^d^	20.61 ± 15.18	15.80 ± 10.45	−4.81 (−11.87, 2.25)	21.61 ± 12.57	21.20 ± 13.98	−0.41 (−5.89, 5.08)	0.133	0.074^b^	0.352	0.029^a^	0.377	0.026^a^

Notes: Values are presented as mean ± standard deviation. Δ, mean change from baseline. Abbreviations: *p* (significance value), *η* ^*2*^ *p* (partial eta squared). Magnitude effect.

^a^ (small).

^b^ (medium).

^c^ (large).

^d^ n = 32 (n = 15 in the control group, n = 17 in the exercise group, due to undetected values).

## 4 Discussion

This study evaluated the effects of 16 weeks of combined exercise training on HRQoL and stress levels in sedentary middle-aged workers. The main finding of the present study was that, compared with the control group, the exercise group significantly improved some domains of quality of life, such as the mental component summary and the social functioning subdomain, after 16 weeks of combined training. Higher magnitudes of improvement were found for the mental component summary and mental health subdomain after the exercise program, which resulted in significant differences between the groups. Furthermore, a higher magnitude of improvement was also observed in the exercise group for the role-emotional and vitality subdomain; however, these changes were not significant. Our results failed to show significant changes in physical component summary and its related subdomains. The exercise group perceived reduced stress levels compared to the control group after the 16 weeks of follow-up; however, this change was not significant. No significant effects were observed for salivary stress biomarkers (i.e., cortisol and alpha-amylase). Although our hypothesis was not fully supported, improving the mental domain of HRQoL is paramount in our sample of middle-aged workers.

Our results are consistent with previous studies (Collins et al., 2021; Pietta-Dias et al., 2019; Tozetto et al., 2022) that demonstrate the effectiveness of combined exercise training in improving the mental component of quality of life among middle-aged and older adults. A previous cross-sectional study published in the *Lancet Psychiatry* also suggested that individuals who exercised 3 to 5 times/week for 30–60 min/session may enhance mental health, reducing mental burden (Chekroud et al., 2018). Likewise, exercise can help relieve several mental disorders, including depression (Harvey et al., 2018) and anxiety conditions (Aylett et al., 2018). In relation to the physical component score and its domains, a slight but not significant decrease in physical role, physical functioning, bodily pain, and the physical component score was observed in the exercise group after 8 weeks of intervention. It is possible that the decrease in bodily pain (indicating more perception of bodily pain) and the other parameters after the 8-weeks of the program is related to resistance training-induced delayed onset of muscle soreness, which is a common effect of few initial training sessions among untrained subjects (Sillanpää et al., 2012) and may persist for 7 days postexercise; later on, muscle soreness usually becomes milder or disappears (Sillanpää et al., 2012; Pearson et al., 2023). The increase in pain perception and decrease in the role-physical assessment by SF-36 have also been previously reported in middle-aged adults by Sillanpää et al. (2012) and Levinger et al. (2007). However, although not significant, a slight increase in physical component and its subdomains were observed for the exercise group after 16 weeks of intervention.

Our results are consistent with a previous study that found that 16 weeks of combined exercise training effectively enhanced the mental component score and its subdomains but not the physical component (except the physical function subdomain) in obese adults (Tozetto et al., 2022). On the other hand, a clinical trial conducted by Collins et al. (2021) found that 8 months of combined exercise training improved both mental and physical component scores, as well as the individual subdomain’s scores of SF-36 among overweight or obese adults at risk of cardiometabolic diseases. Moreover, Baptista et al. (2017) observed that a 24-month multicomponent program (including aerobic, resistance, flexibility, and balance exercises) results in the improvement of three physical subdomains in addition to the physical component score itself but does not improve the mental component score and its subdomains in diabetic older adults. The studies that found significant improvements in the SF-36 physical component applied long-term combined training programs (Collins et al., 2021; Baptista et al., 2017), so it is plausible that the effects of exercise on the physical component are only observed in the long term. In this line, Tozetto et al. (2022) reported that the enhancement of the SF-36 mental component might precede the physical benefits, as they require more stimuli to adapt. In contrast, the increased sense of vigor, the distraction of stressful environments, and belonging to one’s social group are factors that promote psychological wellbeing (Tozetto et al., 2022). According to these authors, the evidence about the effects of combined training interventions on HRQoL physical domain is still inconclusive (Tozetto et al., 2022).

In relation to stress, although not significant, the exercise group tend to improve their perceived stress levels after a 16-week follow-up, while the control group worsened. Moreover, no significant effects were observed for salivary cortisol and alpha-amylase. This trend to decreased perceived stress levels by the exercise group is an important clinical result since psychological stress has a harmful impact on health and wellbeing (O’Connor et al., 2021). Short-term responses to stress include sleep problems (Chen et al., 2023), elevated blood pressure and heart rate, headaches, and unhealthy behaviors such as smoking (Schneiderman et al., 2005). Long-term responses include the increased risk of developing anxiety and depression disorders (Wiegner et al., 2015), cardiovascular diseases (Sara et al., 2018), metabolic syndrome and type 2 diabetes (Harris et al., 2017), and premature mortality (Prior et al., 2016). In the work context, stress and its associated health effects can result in lower productivity from absenteeism and presenteeism and higher healthcare costs (Kuster et al., 2017). A previous study (Silva et al., 2022) in sedentary workers found that the exercise group participants presented lower perceived stress levels after 16 weeks of a home-based combined training program than the control group. Likewise, the same authors did not observe significant changes within groups and differences between groups in cortisol and alpha-amylase levels (Silva et al., 2022). More research is needed to understand the impact of exercise on these markers.

The randomized controlled trial design is one of the strengths of this study. Another important strength was the sample of sedentary middle-aged workers free of major chronic conditions, which reduced the number of potentially eligible participants but increased the representativeness of the HRQoL and stress in this population. Moreover, a supervised exercise intervention designed to meet the total exercise volume recommended by the WHO physical activity guidelines and the optimal adherence to the program are also important strengths. The use of well-validated instruments to assess HRQoL and perceived stress and the 3-times points of assessment is also an important factor.

Nevertheless, some limitations should also be pointed out. Firstly, our sample size calculation was based on the primary outcome of this project (i.e., HOMA-IR), so it is plausible that the lack of an exercise effect on the physical component of HRQoL and stress markers was caused by the study’s small sample size. Secondly, we could not explore subgroup sex differences due to the reduced sample size. Thirdly, the participants’ motivation during the intervention was also not assessed. At the beginning of the intervention, participants were highly motivated; however, the maintenance was challenging in some cases. According to Küüsmaa-Schildt et al. (2019), exercise can improve physical and mental health only when subjects are motivated enough to exercise regularly over a prolonged period. Future controlled studies with large sample sizes should be conducted to better understand the benefits of combined exercise training on the physical component of HRQoL and salivary cortisol and alpha-amylase of stress in middle-aged sedentary workers.

## 5 Conclusion

In summary, we found that 16 weeks of combined aerobic and resistance exercise training can positively influence self-rated HRQoL in previously sedentary middle-aged workers, mainly the mental component summary and the social functioning subdomain. Although not significant, the combined training also promoted a clinically relevant reduction of participants’ perceived stress levels after 16 weeks of exercise training compared to the control group. Our findings suggest that middle-aged sedentary workers will likely benefit from a supervised combined exercise regimen to improve HRQoL (i.e., mental health component summary) and wellbeing.

## Data Availability

The original contributions presented in the study are included in the article/Supplementary Material, further inquiries can be directed to the corresponding authors.
