# Supplementary material for: Effects of combined aerobic-resistance training on health-related quality of life and stress in sedentary adults
Source: Front Aging. 2025 Aug 18;6:1603635. doi: 10.3389/fragi.2025.1603635 (PMC12399556; doi:10.3389/fragi.2025.1603635)
Supplement: Supplementary file 1 [file DataSheet1.pdf]

Supplementary Material

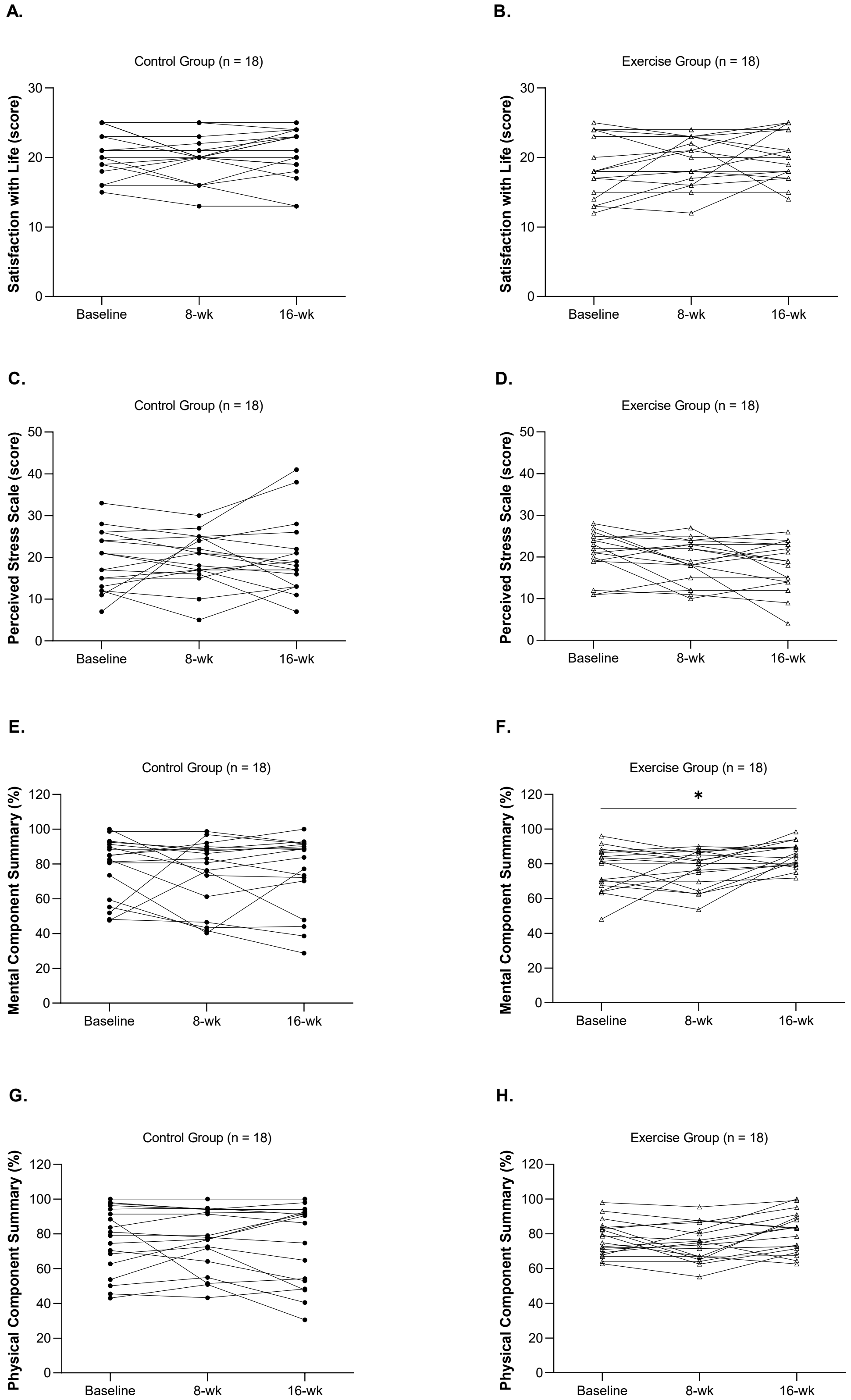

Figure S1. Individual scores on the Satisfaction with Life Scale, Perceived Stress Scale, SF-36 Mental Component Summary and SF-36 Physical Component Summary at baseline, 8 weeks and 16 weeks in middle-aged adults. *A significant improvement was observed in the Mental Component Summary for the exercise group ($X^2_F = 6.113$, $df = 2$, $p = 0.047$, Kendall's W = 0.170); Pairwise comparisons showed significant differences between baseline and 16 weeks (within-group) and between 8 and 16 weeks (within-group).

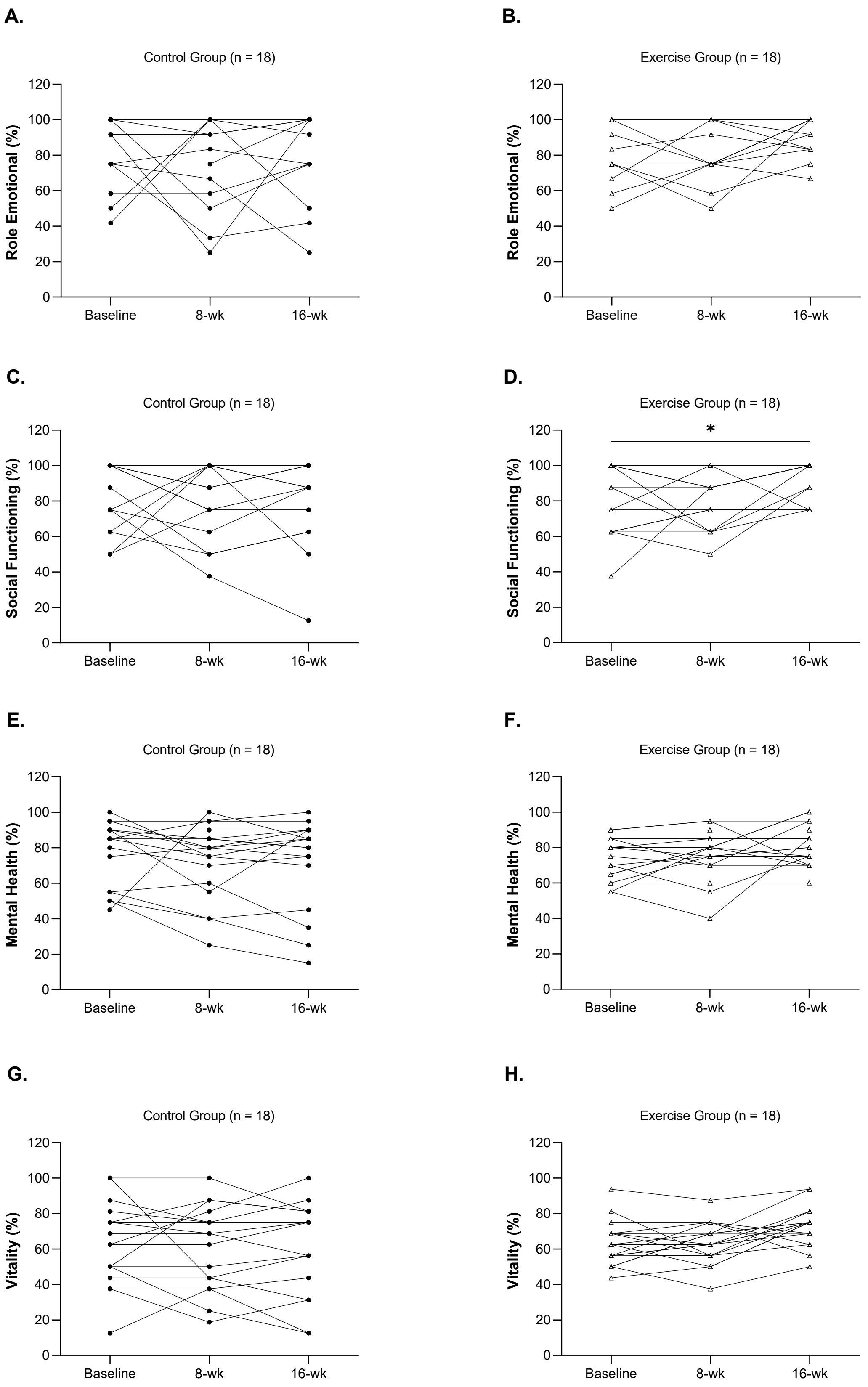

Figure S2. Individual scores on the SF-36 Mental Component Summary Subdomains (Role Emotional, Social Functioning, Mental Health, and Vitality) at baseline, 8 weeks and 16 weeks in middle-aged adults. *A significant improvement in Social Functioning subdomain was observed in the exercise group (X^2_F = 6.450, *df* = 2, *p* = 0.040, Kendall's W = 0.179).

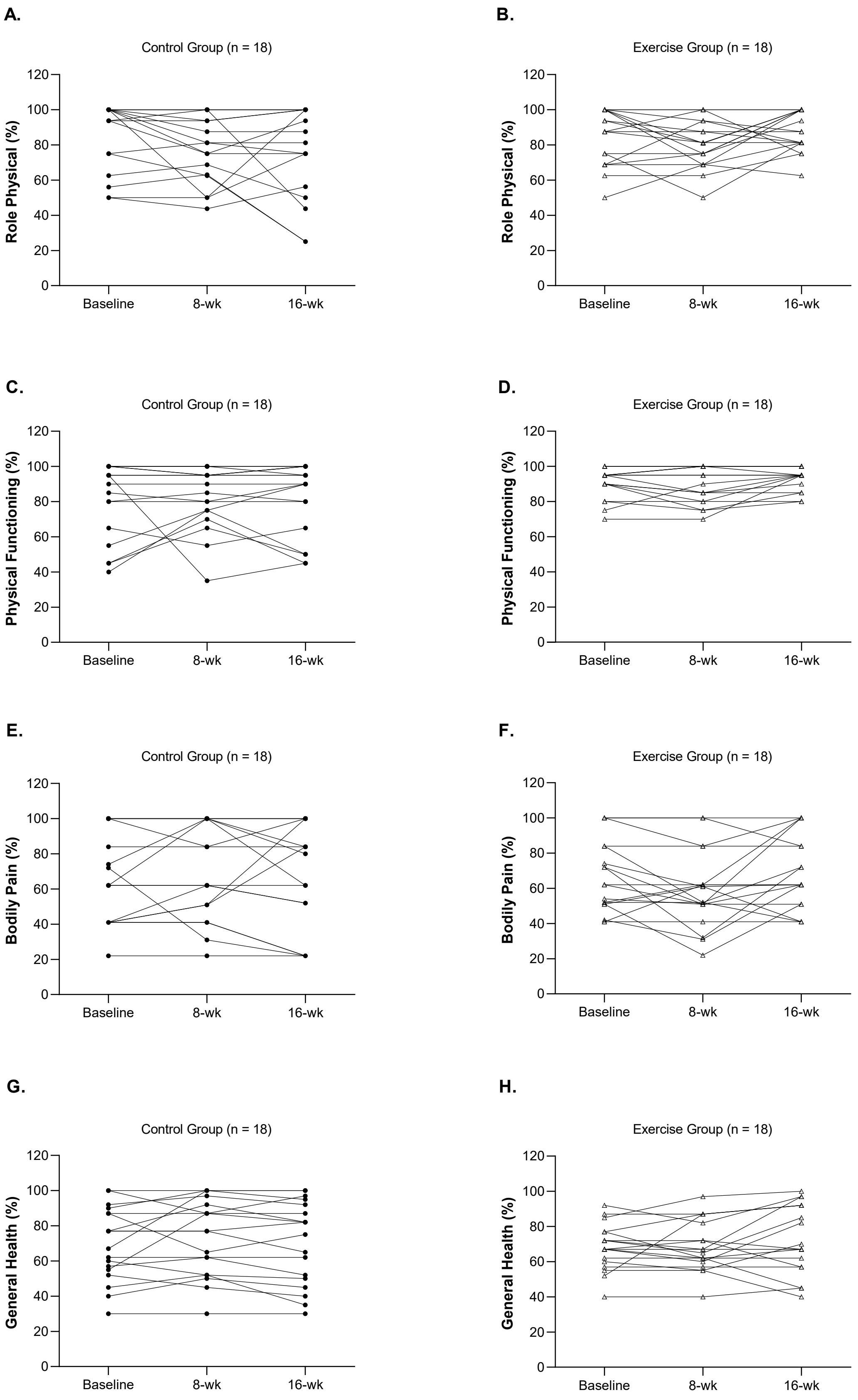

Figure S3. Individual scores on the SF-36 Physical Component Summary Subdomains (Role Physical, Physical Functioning, Bodily Pain, and General Health) at baseline, 8 weeks and 16 weeks in middle-aged adults.
